# Toward an Improved Multi-Criteria Drug Harm Assessment Process and Evidence-Based Drug Policies

**DOI:** 10.3389/fphar.2018.00898

**Published:** 2018-08-20

**Authors:** Veljko Dubljević

**Affiliations:** ^1^Department of Philosophy and Religious Studies, North Carolina State University, Raleigh, NC, United States; ^2^Science, Technology and Society Program, Interdisciplinary Studies Unit, North Carolina State University, Raleigh, NC, United States

**Keywords:** harm assessment, harm reduction, drug policy, multi-criteria drug harm scale, ethical legal and social issues in non-medical drug use

## Abstract

Drug scheduling within the international system of drug control and national legislation has been recently criticized as having insufficient footing in scientific evidence. The legal harms related to non-medical uses of certain drugs (e.g., cannabis) have arguably exceeded their physiological and social harmfulness compared to legally available substances (e.g., tobacco), which prompted some states to explore alternative regulation policies, similar to the drug regime in the Netherlands. Other legally prescribed drugs (e.g., stimulants) created a surge of interest for “better than well” uses, while yet others (e.g., opioids) caused an epidemic of dramatic proportions in North America. The evidence-based multi-criteria drug harm scale (MCDHS) has been proposed as a way of grounding policy in the actual degree of harmfulness of drugs. Indeed, the scale has had great ramifications in several areas of policy, and it has been used extensively in distinct lines of interdisciplinary research. However, some aspects of MCDHS remain disputed. For example, the way the data has been generated has been criticized as suffering from “expert bias.” This article reviews strengths and weaknesses of evidence provided with the use of MCDHS. Furthermore, the author argues that the shortcomings of MCDHS can be resolved by offering methodological improvements. These include (1) dissociating the harms of use from harms of abuse, (2) adding the perspectives of people who use drugs, pharmacists, and general medical practitioners along with the expert assessments, and (3) focusing on subsets of drugs to allow for comparison without mixing different social contexts of drug use. The paper concludes with outlines of substance subset-specific extensions of the MCDHS and related policy proposals in the four areas identified as generating the most controversy: non-medical use of opioids, “study aid” uses of stimulants, shifting trends in nicotine containing products, and regulation of medical and recreational uses of cannabis.

## Introduction

It is safe to assert that the international drug control regime is in flux. The lone example of the Netherlands in terms of loosening the control and decriminalizing or legalizing the use of certain illegal drugs presumed to be safer than previously assumed (e.g., cannabis) has recently been followed by certain states in the U.S., and on the level of national legislation, Uruguay and Canada (Caulkins and Kilmer, 2016; Zhang, 2018). On the other hand, legal drugs such as prescription stimulants and opioids, which have been presumed (and regulated as) safe, have turned out to be quite a challenge in terms of public health. Finally, the promise of “safer forms” of nicotine-containing products has been complicated by new “abuse-like” trends in the use of e-cigarettes. This underscores the need to revise the scientific bases of drug control policies, and most notably, the assessment of harm.

Currently, in most jurisdictions, transnational pharmaceutical corporations are funding the studies that provide evidence that new drugs and substances are safe and effective. Even though this might introduce significant bias, such industry-funded studies are necessary since they are very expensive and governments do not have sufficient funds. However, the vested interests of the pharma industry are not the only source of bias. Namely, most countries and international agencies have drug classification systems that purport to be structured according to the post-market monitoring of the relative risks and dangers of psychoactive substances. Details vary from one jurisdiction to another, but some sort of scheduling classification is in use, based on binding international treaties (i.e., the Single Convention on Narcotic Drugs of 1961 as amended by the 1972 Protocol, the Convention on Psychotropic Substances of 1971, and the United Nations Convention against Illicit Traffic in Narcotic Drugs and Psychotropic Substances of 1988 – see United Nations Office on Drugs and Crime [UNODC], 2018). For instance, in the United States, the federal Controlled Substances Act (CSA) provides the rationale for scheduling in terms of “potential for abuse” and “abuse rate” as “a determinate factor in the scheduling of the drug […] Schedule I drugs have a high potential for abuse and the potential to create severe psychological and/or physical dependence. As the drug schedule changes - Schedule II, Schedule III, etc., so does the abuse potential - Schedule V drugs represent the least potential for abuse” (Drug Enforcement Agency [DEA], 2018).

Within the system of international treaties and drug control policies on a national level, scheduling is an important issue, as it determines how drugs and substances are legally regulated—and more importantly, how users of drugs and substances are treated. For instance, cannabis is federally scheduled in the category of drugs and substances with the most potential for abuse (Schedule I), whereas oxycodone (available as a prescription opioid) and amphetamine (available as a prescription stimulant) are ranked as Schedule II and nicotine containing products are not scheduled. Recently, the scheduling of drugs and addictive substances has been criticized as having insufficient footing in scientific evidence (see Nutt et al., 2007, 2010, see also Fischer and Kendall, 2011). Indeed, some perspectives on the harmfulness of drugs have been informed by ideological—and at times, racist—agendas. For example, the introduction of the key legal controls of drugs (such as the 1914 Harrison Act) was bolstered by playing on public fears of “drug-crazed, sex-mad negroes” that are “murdering whites under the influence of drugs,” along with “degenerate Mexicans” smoking marijuana and “Chinamen seducing white women with opium” (see Cairncross, 2001).

Putting past political rhetoric aside, the issue is not whether policy-makers in ages past have been racist, but whether the socially imposed drug harms they helped enact can be justified today. Many experts believe that they cannot. For instance, Fischer and Kendall (2011) claim that drug and substance control systems have little to no footing in scientific evidence and fail to follow elementary principles informed by empirical logic. According to this view, “drug scheduling [...] originated as a tool of socio-economic control of non-white minority groups, and hence the original drugs included successively in the drug control schedule were opium, cocaine and cannabis (1908–1925). The conceptual framework laid then, and which persists today, *had neither public health, nor pharmacology, nor any attempt of rigorous harm quantification as a foundation*” (Fischer and Kendall, 2011, p. 1891 – emphasis added).

Such criticisms may have motivated certain state jurisdictions in the United States to contradict federal law, at least in terms of cannabis regulation. Indeed, a lengthy debate over drug policy has concluded that prohibitive policies seem to be discredited (see e.g., Duke and Gross, 1993; De Greiff, 1999; Husak, 2005, 2007). Most notably, even staunch supporters of drug control (see e.g., De Marneffe, 2005; Wilson, 2007) agree that the current prohibition regime is too harsh and costly, especially in cases of relatively harmless drugs and substances. However, the problem is how to establish transparent, evidence-based criteria about which drugs and substances are “relatively harmless.”

## The Initial Multi-Criteria Drug Harm Scale Proposal

The multi-criteria drug harm scale (MCDHS) has been proposed as a solution to this problem (Nutt et al., 2007). The idea behind the scale is that qualitative methodology, such as the Delphi method (which entails consulting experts separately to rate drug harms without disclosing the identity of the expert pool in the process) and consensus workshops can provide a platform for a complex and thorough deliberation of the multi-faceted nature of harm from drug and substance use.

According to the original publication detailing the method, a group of United Kingdom-based experts in psychiatry, pharmacology, and addiction used a 4-point scale to rate drugs in three major dimensions of harm: physical health effects, potential for dependence, and social harm, with 0 representing no risk; (1), some risk; (2), moderate risk; and (3), extreme risk (Nutt et al., 2007). Before 16 experts met to discuss and provide the final rankings, a first wave of 29 expert responses was analyzed by the study authors and disseminated to the expert workshop participants. The resulting ranking was the end-product of iterative evaluations based on best available evidence: the final numbers represented mean values from multiple assessments.

This methodology promised to offer a systematic framework and process that could be used by national and international regulatory bodies to assess the harm of current and future drugs that have been used for non-medical purposes. However, the harmfulness ranking of drugs produced by this assessment process differed markedly from the assumptions of most regulatory systems.

Namely, the ranking of harmfulness confirmed some of the expectations expressed in the academic debate on drug control: heroin was ranked as the most harmful drug whereas the harm scores of cannabis were much lower than that of currently legal substances, such as tobacco. However, surprisingly, alcohol was initially rated fairly high on the harmfulness range whereas the “psychedelic drug” LSD and the “party drug” MDMA/ecstasy scored very low. The fact that LSD and MDMA are prohibited and strongly regulated substances while alcohol is legally available caused an uproar of public controversy as a reaction to these harm ratings (Editorial, 2009). Additionally, khat, a less well-known stimulant herb traditionally chewed in Yemen, Somalia and Kenya, was initially ranked as the least harmful substance (see next section).

One of the strengths of the MCDHS was that a process of utilizing large areas of knowledge about drugs and potentially addictive substances has been systematized in a transparent manner, which allows for replication, as well as improvement of the methodology. Indeed, a follow up study in the Netherlands (Van Amsterdam et al., 2010) was published, and the methodology applied by 19 Dutch experts produced similar results: legal substances such as alcohol and tobacco were rated as drastically more harmful than some illegal drugs such as cannabis and MDMA/ecstasy. The correlation coefficient between the two sets of rankings has been calculated at 0.87, indicating a high degree of reliability and validity. The most harmful substances according to this ranking were crack cocaine, heroin, tobacco and alcohol whereas the least harmful were anabolic steroids, khat, LSD and psilocybin/‘magic mushrooms.’ This study also aimed at improving the MCDHS methodology by (1) introducing shared ‘fact sheets’ on drugs to increase transparency and (2) rating harms at individual and population level. Another strength of MCDHS was that harm rankings provided numerical assessments that could be analyzed in different areas of academic research, in order to provide guidance on specific drug policy proposals (see e.g., Dubljević, 2013). However, the public controversy over harmfulness of alcohol (mentioned above) was not isolated – very soon a lively debate ensued about the methodological usefulness of the approach.

## Criticisms and Objections

Important criticisms have been leveled against the MCDHS on methodological grounds and specific concerns raised in the cases of MDMA/ecstasy and khat. In an illuminating article, Parrott (2007) offered extensive criticism of the specific ratings of the initial scale. First of all, Parrott’s analysis revealed that it is probable that some existing drug harms were not perceived by the experts, whereas other harm rankings (such as for alcohol) were driven by outlier populations (e.g., chronic alcohol abusers). For instance, MDMA/ecstasy users report on average 8 physical and 4 psychological problems, which they attribute to their drug use. Also, social drinkers (as opposed to heavy drinkers) are used as healthy control groups in studies of MDMA/ecstasy use (Parrott, 2007). Also, Parrott notes that khat is seldom used in Western societies, whereas certain communities with a cultural tradition of use (mostly in Somalia, Yemen and Kenya, and respective expatriate communities in the West) experience a range of adverse effects, including significant gastro-intestinal distress, with epigastric bloating, abdominal distension and genito-urinary problems. Long term khat-chewing leads to developing oral cancers (similar to tobacco-chewing) and addiction: acute mood gains are followed by adverse withdrawal symptoms, insomnia followed by delayed waking, reduced daily work performance, anorexia, and increased psychiatric distress (Parrott, 2007).

Finally, Parrott states that khat use may be associated with cognitive performance deficits (e.g., 25% of students at a Somali University who were khat chewers had significantly lower academic performance grades, despite coming from higher income families), increased psychosocial distress and financial hardship (e.g., many users in Kenya spend more than half of their domestic budgets on khat).

Thus, it is safe to assume that the lowly rankings of harm for khat and MDMA/ecstasy could be more likely connected to a lack of personal experience (and perhaps relevant data) of the experts in the harmful effects of these specific substances rather than genuine lack of harm. Additional issues that MCDHS failed to address (and was criticized for) are lack of attention to situational factors (see Caulkins et al., 2011), value judgments (see Kalant, 2010), and input from relevant stakeholders (see Forlini et al., 2013).

As Nutt (2011) rightly notes in his response to critics, the fact that a certain methodology has drawbacks does not mean that it should be entirely abandoned, especially if no alternative has been proposed. However, these important criticisms that have been leveled against the MCDHS on methodological grounds, and specific concerns raised in the cases of MDMA/ecstasy and khat require a critical assessment of the methodology as well as determination of how far the weaknesses of the scale affect the conclusions drawn from it, most notably the policy proposals.

## Updated Methodology: Multi-Criteria Decision Analysis for Evidence-Based Drug Harm Assessment and Policy

In an effort to increase validity of the scale, the lead proponent (David Nutt) founded the Independent Scientific Committee on Drugs in the United Kingdom and repeated the drug harm ratings with a revised methodology based on weighted scores of multi-criteria decision analysis (MCDA) modeling (see Nutt et al., 2010). This introduced the expert assessment not only to raw harmfulness scores but also to relevant importance (or weights) of different harm dimensions. Namely, drugs were scored with points from 0 to 100, with 100 being assigned to the most harmful drug on a specific criterion, and 0 indicating no harm on that particular criterion. Weighting subsequently compared the drugs that scored 100 across all the criteria, thereby expressing the value judgment that some criteria are more important than others (see Nutt et al., 2010). The final list of criteria included Drug-specific mortality, Drug-related mortality, Drug-specific damage, Drug-related damage, Dependence, Drug-specific impairment of mental functioning, Drug-related impairment of mental functioning, Loss of tangibles, Loss of relationships, Injury (to others), Crime, Environmental damage, Family adversities, International damage, Economic cost, and Community (cohesion/reputation). These criteria, with minor alterations to suit the context, have been used in most follow up studies (discussed below).

As with the Netherlands study (Van Amsterdam et al., 2010), the repeated ranking for the UK assessed harms to users (the first nine criteria) and harms to others (the latter seven criteria) and found that alcohol, heroin, crack cocaine and methamphetamine are the most harmful drugs, whereas the least harmful were MDMA/ecstasy, LSD, buprenorphine (an opioid replacement drug) and psilocybin /‘magic mushrooms’. The harms of alcohol “to others” appear to have disproportionally affected the rating and precipitated the heated exchange in the journal Addiction, where some of the critics argued that the “false promise” of an evidence-based policy was ultimately rooted in “false premises” of a deeply flawed methodology (see Caulkins et al., 2011).

The lead authors engaged in the two national MCDHS studies (in the United Kingdom and Netherlands, respectively) appeared to be undaunted by the controversy; they joined forces and expanded their scope by creating a Europe-wide expert panel for assessment of drug harms by using the MCDA modeling methodology (see Van Amsterdam et al., 2015a). The stated goal was to mitigate the fact that a certain drug might be scarcely used in one region of Europe, whereas it is highly used in another region. Again, the rating found that alcohol, heroin, crack cocaine and methamphetamine are the most harmful drugs, whereas the least harmful were anabolic steroids, LSD, buprenorphine and psilocybin. The proponents of the methodology also tried to accommodate some of the deserved criticism in subsequent work by incorporating assessments of well specified substances containing nicotine (see Nutt et al., 2014), prescription opioids used in the United Kingdom (Van Amsterdam et al., 2015b), and even by modeling harms of specific drug policies for regulation of alcohol and cannabis (Rogeberg et al., 2018).

The study on the harmfulness of nicotine-containing products developed the rating that found cigarettes as most harmful nicotine containing products (100%), followed by small cigars (67%), pipes (22%) and cigars (16%). The least harmful nicotine containing substances were found to be Electronic Nicotine Delivery Systems or ENDS (5%), nasal sprays (3%), Oral Nicotine Delivery Products (2%), and Dermal Nicotine Delivery Products or “patches” (1%). Even though there is little doubt that the three nicotine containing products ranked as least harmful are much safer than traditional tobacco products, there remain doubts whether all ENDS are really safe or if data about their long term harmfulness is simply lacking (see e.g., Kim et al., 2015). Similarly, there are reasons to question whether the sharp drop in harmfulness ratings between small cigars and pipes is more due to lower prevalence of use rather than genuine lack of long-term adverse effects, which would indicate that at least some methodological artifacts are embedded in the research findings (see the discussion on improvements of the methodology below). An additional confound is engendered in the interpretation of data. Does the fact that pipes are rated as more than four times safer than cigarettes, or that ENDS are rated as twenty times safer, mean that public health messages or regulation should incorporate these findings and argue for a change in preferred product in long term nicotine users? Some of the proponents of the MCDHS believed so (most notably David Nutt) and petitioned the Australian Government to change the current scheduling of nicotine containing products if they are ENDS. The petition ultimately failed, but in the public deliberations, expert opinions of the proponents of MCDHS were acknowledged and contrasted to worries that “dripping” – ultimately a drug abuse technique – may be leading to exposure to high nicotine levels (Australian Government Therapeutic Goods Administration, 2018).

As was the case with the controversy over policy recommendations regarding alcohol (see Nutt et al., 2010; Kalant, 2010, Caulkins et al., 2011; Nutt, 2011), there are good reasons to consider applying caution in translating research findings directly into policy; the historical record of the failed policy of prohibition in the United States, along with counter-intuitiveness of many findings from the MCDHS/MCDA process, has made many policy makers and commentators reluctant to take heed of the policy recommendations engendered by the methodology. However, there are areas where evidence-based interventions and policy evaluations are desperately needed, at least in the North American context, and these are with opioid and cannabis regulation. As noted above, the MCDA methodology has provided some valuable input, and the transparent nature of the process allows for methodological improvements.

The study on the harmfulness of non-medically used prescription opioids (in the United Kingdom) developed the rating that found injected heroin as most harmful (99%), followed by smoked heroin (70%), fentanyl (55%), and diamorphine (50%). The least harmful prescription opioids were found to be tramadol (16%), suboxone (15%), compound codeine products (12%) and codeine (10%). A surprising finding was that oxycodone was rated as only moderately harmful (22%), even less harmful than methadone (30%). The study authors note on that particular issue that their harmfulness ratings are “not independent of prevalence of use” (Van Amsterdam et al., 2015b, p. 1003) and that the aggressive marketing and promoting of oxycodone in the U.S. could mean that this same substance could be much more dangerous in other populations. Direct to consumer marketing of opioids in the United States could be an additional aspect of social harm that future studies might need to address.

In terms of assessing policy options, MCDA methodology was applied as a new approach to formulate and appraise regulations of alcohol and cannabis (see Rogeberg et al., 2018). It is hard to determine what effect (if any) this new application of the methodology might have for the future of public policy, but it is fair to assert that the transparency of assessment criteria at least invites informed public debate. The list of 27 criteria grouped into seven thematic clusters included Health (Harm reduction to users, Harm reduction to others, Shift to lower-harm products, Encouraging treatment, Improving product quality), Social (Promoting drug education, Enabling medical use, Promoting research, Protecting human rights, Promoting individual liberty, Improving community cohesion, Promoting family cohesion), Political (Supporting international development or security, Reducing industry influence), Public (Promoting well-being, Protecting the young, Protecting the vulnerable, Respecting religious or cultural values), Crime (Reducing criminalization of users, Reducing acquisitive crime, Reducing violent crime, Preventing corporate crime, Preventing criminal industry), Economic (Generating state revenue, Reducing economics costs), and Costs (Policy introduction costs, Policy maintenance costs).

The four policies rated were free market/‘laissez faire,’ state control, decriminalization, and absolute prohibition. Expert ratings favored state control for both alcohol and cannabis regulation, whereas absolute prohibition was least favored. The major difference was that absolute prohibition of alcohol did garner some support (55%) from experts based on the identified clusters of criteria, whereas absolute prohibition of cannabis garnered minimal support (5%). Again, it is too early to tell if these assessments might guide or are the result of the ongoing ‘flux’ in drug policy proposals, but undeniably, past regulatory approaches have been motivated by ‘extreme solutions’ that failed to address the full set of relevant issues (see Rogeberg et al., 2018).

The MCDHS/MCDA methodology appears to have provided at least some robust results in terms of reliability and validity and allows for breaking down complex evaluations into a series of smaller, more easily assessed issues. However, many methodological issues remain unresolved (see next section), and the question of objective replicability looms large. Namely, although the proponents of the methodology boast of a high overall correlation in follow-up replication studies, these are not independent studies; key proponents of the model have been active in the studies, and most of the time, only European experts have been involved in the ratings. Thus, independent replication is still lacking. Indeed, as Van Amsterdam and colleagues have noted, “it would be of interest to repeat [the] MCDA harm assessment in the United States,” (Van Amsterdam et al., 2015b, p. 1003) but the scientific and public interests would best be served if this was done using an improved methodology and by unrelated researchers in North and South America and followed up by similar assessments elsewhere in the world.

## Remaining Methodological Artifacts and Blind-Spots

The major obstacle to the adequate assessment of harm is the reification of substance harms without allowing for the differential recognition of context of use. Namely, the greatest controversy surrounded the classification of alcohol as the most harmful substance in the European rating of drug harms (Van Amsterdam et al., 2015a). This result undermined the face validity of the methodology and at the same time offers clues as to how the method can be improved. The relatively high incidence of fatal drunk-driving accidents and health issues connected to long-term alcohol abuse have pushed the harmfulness rating for alcohol over those of heroin and crack cocaine. Yet, social drinkers (as used as control groups in drug abuse studies) can and do drink responsibly. The point is not that alcohol is not potentially dangerous or that there aren’t harms associated even with “responsible use” of alcohol, but that the social context of use matters. For instance, hydration is essential for survival, and at the same time, overuse of hydration can be deadly (see Ballantyne, 2007). This underscores the need to provide multiple assessments for harm: harms of regular, low-dose use, and harms of overuse and abuse.

Furthermore, it is clear that the alleged “universal knowledge” of experts has specific blind spots, most notably in terms of substances where they lack personal experience. This is where the “local knowledge” of people who use these drugs might be beneficial in correcting the “expert bias.” Certainly, science often outstrips common sense experience and is essential for identifying phenomena that are not immediately perceptible to people (for a longer argument, see Briggle and Mitcham, 2012). However, consulting expert opinions of scientists does not guarantee that assessments will be correct, especially with phenomena that have an important social component. For example, psychiatry experts consulted prior to the Stanford prison experiment and the Milgram experiments not only failed to predict the extent to which negative behaviors would take place, but their expert opinions were so widely off the mark so as to designate some of the most harmful psychological experiments in known history as harmless (see Zimbardo, 2008). Therefore, additional safeguards for objectivity and stakeholder perspectives are necessary to provide relevant input in the assessment procedure, and any assessment results need to be acknowledged as provisional and revisable based on new evidence. In this respect, adding the perspectives of people who use drugs, pharmacists, and general medical practitioners along with the expert assessments might need to be the new ‘gold standard’ for substance harm assessment.

Finally, the policy analysis of different potentially addictive substances should take into account the source of the substance in question. For instance, a policy analysis of stimulants might need to have at least two separate harm assessments for many of the drugs in question. Namely, taking into consideration the differences between say prescription amphetamines (such as Adderall, the purity of which is controlled) and street amphetamines (to which additional harmful substances are often added) seems to be a *conditio sine qua non* of successful application of the methodology.

## The Way Forward

Having a transparent methodology for providing an evidence base for drug policy which still needs to be perfected is better than having no methodology at all. After the careful analysis of the strengths and weaknesses of the MCDHS/MCDA methodology, it is important to identify areas of drug/substance harms that need to be urgently (re)assessed. Given that the current major issues with the public health concerns are related to increased opioid and stimulant use, shifts in nicotine use, and cannabis regulation, it makes sense to make the assessment of harms in these categories with the improved methodology a priority.

The reassessment of opioids might provide valuable data that at the same time validates and expands on the previous results. Namely, it could be the case that the moderate harmfulness rating of oxycodone is actually correct in assessing harms of regular, low dose use, whereas the separate harmfulness ranking of non-medical use might provide decision makers and physicians with valuable evidence that would guide better crafted drug policies. Furthermore, dissociating between the harms (e.g., in terms of Drug-specific mortality) of the substance in question from different sources and grades of purity might provide input for harm reduction strategies that do not cause undue social and legal burdens. Most notably, these should not be based on expert assessments alone, but informed by perspectives from people who use drugs, pharmacists, and general medical practitioners. Multiple iterative rankings prior to final expert ranking should be the ‘gold standard’ in harm assessments. Finally, expanding the list of criteria to include the perception of certain benefits of regular, low dose use (e.g., Promoting well-being in populations experiencing chronic or acute pain) and perception of specific social factors (e.g., Social influence of the industry) might broaden the knowledge pool that informs expert assessments. These suggestions are only tentative, and it is up to multiple groups of workshop participants to make value judgments in terms of inclusion and relative relevance of any single criterion.

Similarly, the assessment of stimulants might provide an important addition to the debate over an epidemic that is happening in the shadow of the opioid crisis: the misuse of prescription stimulants in populations of researchers (see e.g., Maher, 2008), employees (Dubljević, 2012), and students (DeSantis et al., 2008). Prior policy proposals have been taking into account the results from the initial scale ranking (see e.g., Dubljević, 2013), but increased awareness about harmfulness of amphetamines has led to a shift in preference among some student populations and even medical practitioners to newer, atypical stimulant drugs such as modafinil (see Dubljević and Ryan, 2015; Dubljević, 2016). The fact that newer drugs with stimulant-like properties were not rated at all in any of the MCHDS studies significantly limits the debate on evidence based policy options for this class of drugs. Herbal stimulants with amphetamine-like effects such as khat should be reassessed and contrasted not to a generalized list of drugs, but in a narrowed down scope, to similar substances. Furthermore, dissociating between the harms (e.g., in terms of Dependence) of the substance in question from different sources and grades of purity might provide input for public policies that target specific avenues of drug diversion, which might relieve undue social and legal burdens (e.g., should a student that shared their prescription ADHD medication be treated as a drug dealer?). This analysis would also need to explicitly distinguish between safe recreational users of stimulants and unsafe recreational abusers of stimulants, and it would need to include task-specific benefits for users of stimulants (e.g., in terms of “cognitive enhancement” – see Dubljević, 2015; Dubljević et al., 2015). Additionally, the criteria need to include explicitly social benefits of use (e.g., Respecting cultural values in khat use). Although any results of the MCDHS/MCDA methodology have to be interpreted with caution, these ratings are the only available and feasible measure of the safety profiles of stimulants, and as such will benefit multiple societies (see Jotterand and Dubljević, 2016).

The reassessment of nicotine containing products could likewise provide valuable data that at the same time validates and expands on the previous results. Adding the perspectives of (past) users, e-cigarette retailers, and physicians could clarify whether the harms of pipe use and abuse are drastically lower than harms of cigarette use and abuse in terms of specific health outcomes as they are subjectively perceived, and at the same time, they could provide an insight into potential targets for public education and current gaps in evidence. For instance, the current expert rating might have properly rated harms of “cigarette abuse” and “pipe use,” thereby confounding their actual harmfulness. Similarly, differentiating forms of use and abuse of ENDS or e-cigarettes (see Kim et al., 2015) might clarify the conflicting expert opinions in terms of ENDS policy (see Australian Government Therapeutic Goods Administration, 2018). Additionally, probing the perceived social influence of traditional tobacco vs. e-cigarette industry and retailers might benefit the informed public discussions on appropriate harm reduction and discourage use policies and inform targeted taxation efforts that avoid the pitfalls of “regulatory capture.”

Finally, a reassessment of cannabis policies should incorporate a comparative study not only of policy types, but also of specific existing policies (including those at the national level, such as the Netherlands, Uruguay, and Canada, and at the State level such as Colorado, Vermont, California, etc.) along with known Health, Social, Political, Public, Law enforcement, and Economic effects. The inclusion of additional perspectives (i.e., people who grow and/or use cannabis and treating physicians) would drastically reduce bias in this volatile and value laden debate. Explicitly distinguishing between recreational uses of cannabis and recreational abuses of cannabis and including specific medical benefits for certain populations (in terms of e.g., chronic pain management) would provide a better evidence base for effective evaluation of public policy.

In conclusion, researchers have a duty to take heed of methodological improvements and initiate assessments of harms within diverse communities and expert groups. At the same time, decision makers and regulators need to better acknowledge and fund such efforts in order to discharge their mandate to the public. Ultimately, further discussion is needed in order to generate as many proposals for evidence based methodological approaches and specific models of drug harm assessment as possible. This is a necessary step in order to provide desperately needed evidence for legislators at federal, state and local levels, prosecutors making decisions in cases of illegal use of drugs and citizens engaged in drug policy change via initiative and referenda activities (see McBride and Terry-McElrath, 2016). Drugs and potentially addictive substances will be adequately regulated only as a result of a public discussion on a sufficiently large, eligible set of evidence based harm assessments and policy options.

## Author Contributions

The author confirms being the sole contributor of this work and approved it for publication.

## Conflict of Interest Statement

The author declares that the research was conducted in the absence of any commercial or financial relationships that could be construed as a potential conflict of interest.
